# Increased Osmolarity in Biofilm Triggers RcsB-Dependent Lipid A Palmitoylation in *Escherichia coli*

**DOI:** 10.1128/mBio.01415-18

**Published:** 2018-08-21

**Authors:** Magdalena Szczesny, Christophe Beloin, Jean-Marc Ghigo

**Affiliations:** a Institut Pasteur, Genetics of Biofilms Laboratory, Paris, France; University of Washington

**Keywords:** biofilm, LPS, Rcs envelope stress, lipid A, palmitoylation

## Abstract

Biofilms are often described as protective shelters that preserve bacteria from hostile surroundings. However, biofilm bacteria are also exposed to various stresses and need to adjust to the heterogeneous physicochemical conditions prevailing within biofilms. In Gram-negative bacteria, such adaptations can result in modifications of the lipopolysaccharide, a major component of the outer membrane characterized by a highly dynamic structure responding to environmental changes. We previously showed that Gram-negative biofilm bacteria undergo an increase in lipid A palmitoylation mediated by the PagP enzyme, contributing to increased resistance to host defenses. Here we describe a regulatory pathway leading to transcriptional induction of *pagP* in response to specific conditions created in the biofilm environment. We show that *pagP* expression is induced via the Rcs envelope stress system independently of the Rcs phosphorelay cascade and that it requires the GadE auxiliary regulator. Moreover, we identify an increase in osmolarity (i.e., ionic stress) as a signal able to induce *pagP* expression in an RcsB-dependent manner. Consistently, we show that the biofilm is a hyperosmolar environment and that RcsB-dependent *pagP* induction can be dampened in the presence of an osmoprotectant. These results provide new insights into the adaptive mechanisms of bacterial differentiation in biofilm.

## IMPORTANCE

The development of the dense bacterial communities called biofilms creates a highly heterogeneous environment in which bacteria are subjected to a variety of physicochemical stresses. We investigated the mechanisms of a widespread and biofilm-associated chemical modification of the lipopolysaccharide (LPS), a major component of all Gram-negative bacterial outer membranes. This modification corresponds to the incorporation, mediated by the enzyme PagP, of a palmitate chain into lipid A (palmitoylation) that reduces bacterial recognition by host immune responses. Using biochemical and genetic approaches, we demonstrate that a significant part of biofilm-associated lipid A palmitoylation is triggered upon induction of *pagP* transcription by the hyperosmolar biofilm environment. *pagP* induction is regulated by RcsB, the response regulator of the Rcs stress response pathway, and is not observed under planktonic conditions. Our report provides new insights into how physiological adaptations to local biofilm microenvironments can contribute to decreases in susceptibility to antimicrobial agents and host immune defenses.

## INTRODUCTION

In constantly changing environments, bacteria have evolved different mechanisms enabling them to sense and respond to nutrient and stress variations. This capacity for adaptation is particularly important during the development of highly heterogeneous biofilms, in which bacteria are exposed to a variety of physicochemical gradients rarely encountered by free-living organisms (1). Physiological adaptation to the biofilm environment leads to characteristic decreased susceptibility to antimicrobial agents and host immune defenses (2, 3) at the origin of chronic and device-related biofilm infections (4).

Several studies showed that modifications in lipopolysaccharide (LPS) structure in Gram-negative bacteria also contribute to biofilm tolerance of antimicrobial compounds (5, 6). Consistently, we had previously reported that biofilm formation in Escherichia coli and other Gram-negative bacterial species is associated with increased incorporation of a palmitate acyl chain into the LPS lipid A domain. Lipid A palmitoylation attenuates the Toll-like receptor 4 (TLR4)-mediated inflammatory host response and increases *in vivo* survival of biofilm bacteria (7). Lipid A palmitoylation is mediated by the palmitoyl transferase PagP (8–10) and is increased in both biofilm and prolonged planktonic cultures in late stationary phase (7), suggesting that these two environments could share common conditions triggering this modification. However, while E. coli *pagP* expression is repressed by H-NS and positively regulated by the anti-H-NS factor SlyA, the exact mechanism underlying biofilm-associated increased lipid A modification remains unclear.

In planktonically grown E. coli, the *pagP* gene has been demonstrated to depend on the PhoPQ and EvgAS transcriptional regulatory systems (11, 12). The PagP enzyme itself can also be activated posttranslationally by a redistribution of phospholipids within the outer membrane (11). Here we showed that increased lipid A palmitoylation in biofilm results from two distinct molecular mechanisms: a posttranslational activation of PagP observed in biofilm and under late-stationary-phase conditions (12–14) and a previously undescribed transcriptional induction of *pagP* expression in biofilm only. We determined that induction of *pagP* expression in mature biofilm is dependent on RcsB, a regulator of the Rcs envelope stress response phosphorelay (15, 16). This Rcs-dependent induction of *pagP* expression can be recapitulated by exposing planktonic bacteria to high saline stress, suggesting that *pagP* expression is triggered in response to specific physicochemical cues developing within mature biofilms. This report therefore provides new insights into how physiological adaptations to the local biofilm microenvironment can contribute to increased stress resistance in biofilm bacteria.

## RESULTS

### *pagP* transcription is specifically induced in E. coli biofilms.

**FIG 1:**
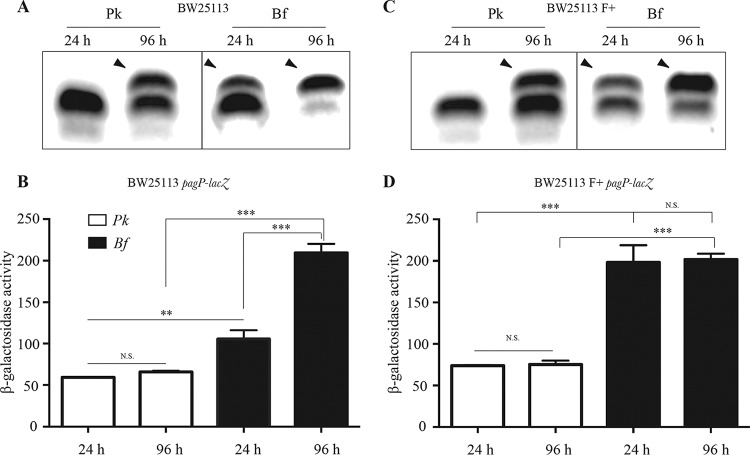
*pagP* transcription is induced in E. coli biofilms. (A and C) Tricine SDS-PAGE/periodate-silver staining analysis of LPS extracted from planktonic (Pk) and biofilm (Bf) E. coli K-12 BW25113 (A) or its closely related derivative BW25113 F^+^ carrying the biofilm-promoting F conjugative plasmid (C). LPS was analyzed after 24 or 96 h of growth in M63B1 supplemented with 0.4% glucose; arrowheads indicate a modified LPS band. (B and D) The corresponding level of *pagP* expression in planktonic (Pk) cultures and biofilm (Bf) was assessed by measuring β-galactosidase activity (Miller units) in E. coli K-12 BW25113 *pagP lacZ* (B) and E. coli K-12 BW25113 F^+^ *pagP*-*lacZ* (D). Statistical significance was assessed using one-way analysis of variance (ANOVA) followed by Bonferroni’s *post hoc* comparison tests. Compared values that are significantly different are indicated by asterisks as follows: **, *P* < 0.01; ***, *P* < 0.001. N.S., not significant.

We previously reported that E. coli biofilm bacteria show a higher level of lipid A palmitoylation on LPS gels than planktonic bacteria. However, this difference between planktonic and biofilm LPS profiles is attenuated with time (Fig. 1A). Since accumulation of lipid A palmitoylated species can result either from posttranslational activation of PagP (12, 13) or from transcriptional induction of the *pagP* gene (17), we decided to monitor *pagP* expression in E. coli using a *pagP-lacZ* transcriptional operon fusion, in which *lacZ* is inserted downstream of an intact *pagP* gene. In contrast to planktonic and biofilm lipid A profiles on LPS gels, we observed a strictly biofilm-associated increase of the *pagP* expression level, whether tested at 24 or 96 h (Fig. 1B). To further confirm the correlation between biofilm formation and transcriptional induction of *pagP* expression in biofilm, we compared the levels of *pagP* expression in BW25113 and MG1655 E. coli K-12 backgrounds carrying or not the biofilm-promoting F conjugative plasmid (18). In both cases, F^+^ biofilm-forming strains displayed earlier maximum induction of *pagP* (Fig. 1C and D; see also Fig. S1 in the supplemental material), confirming the biofilm-associated induction of *pagP* expression. In the rest of this report, *pagP* expression levels are compared at 48 h using F-carrying strains.

10.1128/mBio.01415-18.1 FIG S1  *pagP* biofilm induction in E. coli K-12 BW25113 and MG1655. Comparisons of *pagP* expression levels in E. coli K-12 BW25113 F^+^ *pagP*-*lacZ* and E. coli K-12 MG1655 F^+^ *pagP*-*lacZ* grown under planktonic (Pk) or biofilm (Bf) conditions were performed. Due to the presence of the biofilm-promoting F conjugative plasmid, the time of growth was reduced to 48 h. β-Galactosidase activity was measured after 1 h of incubation. Statistical significance was assessed using one-way analysis of variance (ANOVA), followed by Bonferroni’s *post hoc* comparisons (***, *P* < 0.001). Download FIG S1, PDF file, 0.1 MB. Copyright © 2018 Szczesny et al. 2018 Szczesny et al. This content is distributed under the terms of the Creative Commons Attribution 4.0 International license.

### Transcriptional regulator RcsB regulates *pagP* expression in biofilms.

**FIG 2:**
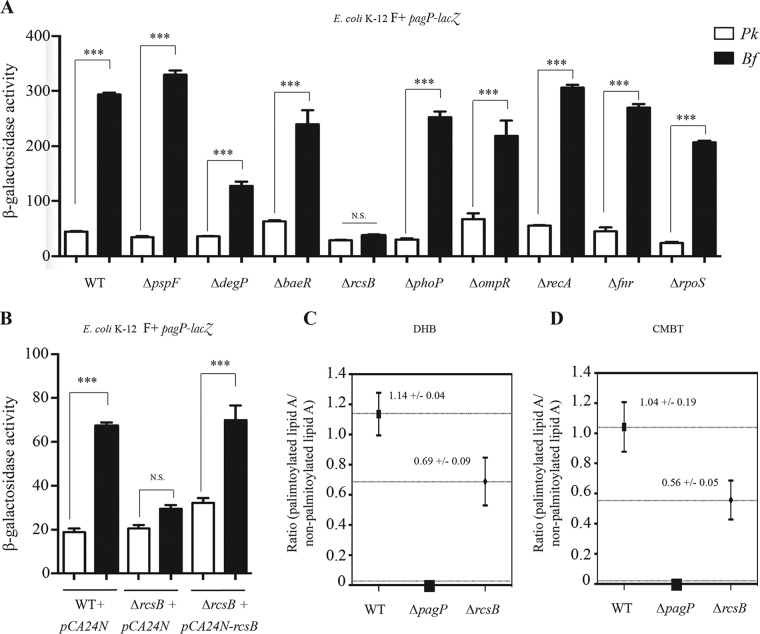
RcsB-dependent regulation of *pagP* increases lipid A palmitoylation in biofilm bacteria. (A) E. coli K-12 BW25113 F^+^ *pagP*-*lacZ* strains, including the wild-type (WT) strain or strains with the *pspF*, *degP*, *baeR*, *rcsB*, *phoP*, *ompR*, *recA*, *fnr*, or *rpoS* gene deleted, were grown in planktonic cultures (Pk) and biofilm (Bf) for 48 h, and β-galactosidase activity was measured. (B) E. coli K-12 BW25113 F^+^ *pagP*-*lacZ* pCA24N, E. coli K-12 BW25113 F^+^ Δ*rcsB pagP*-*lacZ* pCA24N, and E. coli K 12 BW25116 F^+^ Δ*rcsB pagP*-*lacZ* pCA24N-*lacZ* were grown in planktonic (Pk) cultures and biofilm (Bf) for 48 h in M63B1 minimal medium supplemented with 0.4% glucose and 0.05 mM isopropyl-β-d-thiogalactopyranoside (IPTG), and β-galactosidase levels were measured. Statistical significance was assessed using one-way analysis of variance (ANOVA) followed by Bonferroni’s *post hoc* comparison tests (***, *P* < 0.001; N.S., not significant). (C and D) MALDI-TOF MS analysis of lipid A extracted from E. coli K-12 MG1655 F^+^, E. coli K-12 MG1655 F^+^ Δ*pagP*, and E. coli K-12 MG1655 F^+^ Δ*rcsB* 48h biofilm bacteria was performed using a DHB matrix (C) or a CMBT matrix (D). Analysis of lipid A by MALDI-TOF MS in biofilm bacteria shows two major peaks, at *m*/*z* =1,797.4, corresponding to a hexa-acylated nonpalmitoylated lipid A species, and at *m*/*z* =2,035.6, corresponding to a hepta-acylated palmitoylated lipid A species. Results are presented as the ratio of palmitoylated and nonpalmitoylated lipid A species: A(2035.8)/A(1797.4).

*pagP* expression is controlled by histone-like protein repressor H-NS and the SlyA regulator (7). However, while inactivation of *slyA* reduced *pagP* expression in both planktonic culture and biofilms, it did not abolish *pagP* induction in biofilms, suggesting that, in addition to SlyA, other regulators play a role in biofilm-associated *pagP* expression (7). We therefore tested the contribution of various envelope stress and general response regulators (Fig. 2A; see also Fig. S2) and found that a mutation in *rcsB* was the only mutation abolishing all differences between planktonic and biofilm *pagP* expression in a biofilm-forming E. coli strain carrying the *pagP-lacZ* fusion (Fig. 2A). RcsB is the response regulator of the Rcs system, a phosphorelay transduction system that responds to various environmental stresses (19–21). This phenotype was fully complemented upon plasmid-based expression of *rcsB* in medium-copy-number plasmid pCA24N-*rcsB* (Fig. 2B), confirming the contribution of RcsB to *pagP* biofilm regulation. Finally, we investigated the contribution of RcsB transcriptional induction to total accumulation of palmitoylated lipid A species. Using matrix-assisted laser desorption ionization–time of flight mass spectrometry (MALDI-TOF MS), we compared the relative abundances of hexa-acylated (nonpalmitoylated) and hepta-acylated (palmitoylated) lipid A species in E. coli wild-type (WT) and Δ*rcsB* biofilms. The MALDI-TOF MS analysis shows two major peaks, at *m*/*z* =1,797.4, corresponding to the hexa-acylated nonpalmitoylated lipid A species, and at *m*/*z* =2,035.6, corresponding to the hepta-acylated palmitoylated lipid A species. These results show that inactivation of *rcsB* leads to a 2-fold decrease in the ratio of palmitoylated and nonpalmitoylated lipid A species [i.e., lipid A(2035.8)/lipid A(1797.4)] (Fig. 2C and D). To further confirm that the decreased level of palmitoylated lipid A species in the RcsB mutant was due to the absence of *pagP* transcriptional induction, we tested whether deletion of *rcsB* could also impact PagP at the posttranslational level. To address this issue, we used a *Pcl-pagP* strain, in which *pagP* is placed under the control of a constitutive promoter, and monitored the lipid A palmitoylation profile in the presence or absence of RcsB. We did not observe any decrease of lipid A palmitoylation in the *rcsB* mutant (Fig. S3). Taken together, these results indicate that lipid A palmitoylation occurs both through posttranslational activation of the basal PagP enzyme inserted in the outer membrane and through a RcB-dependent transcriptional induction of the *pagP* gene.

10.1128/mBio.01415-18.2 FIG S2  Impact of deletion of regulators on *pagP* biofilm induction. E. coli K-12 BW25113 F^+^ *pagP*-*lacZ* strains, WT or with the *cpxR*, *soxR*, or *luxS* (A) or *evgA* or *relA* (B) gene deleted, were grown under planktonic (Pk) or biofilm (Bf) conditions for 48 h. β-Galactosidase activity was measured. Statistical significance was assessed using one-way analysis of variance (ANOVA) followed by Bonferroni’s *post hoc* comparison tests (***, *P* < 0.001). Download FIG S2, PDF file, 0.1 MB. Copyright © 2018 Szczesny et al. 2018 Szczesny et al. This content is distributed under the terms of the Creative Commons Attribution 4.0 International license.

10.1128/mBio.01415-18.3 FIG S3  Deletion of *rcsB* does not impact PagP at the posttranslational level. Tricine SDS-PAGE/periodate-silver staining analysis of LPS extracted from planktonic E. coli K-12 MG1655 F^+^ *ΔpagP*, MG1655 F^+^ *PcL-pagP*, and MG1655 F^+^ *PcL-pagP* Δ*rcsB* LPS was performed using exponentially growing cells in M63B1 supplemented with 0.4% glucose; arrows indicate a modified LPS band. Download FIG S3, PDF file, 0.1 MB. Copyright © 2018 Szczesny et al. 2018 Szczesny et al. This content is distributed under the terms of the Creative Commons Attribution 4.0 International license.

### RcsB-dependent biofilm regulation does not require RcsACDF or phosphorylation.

**FIG 3:**
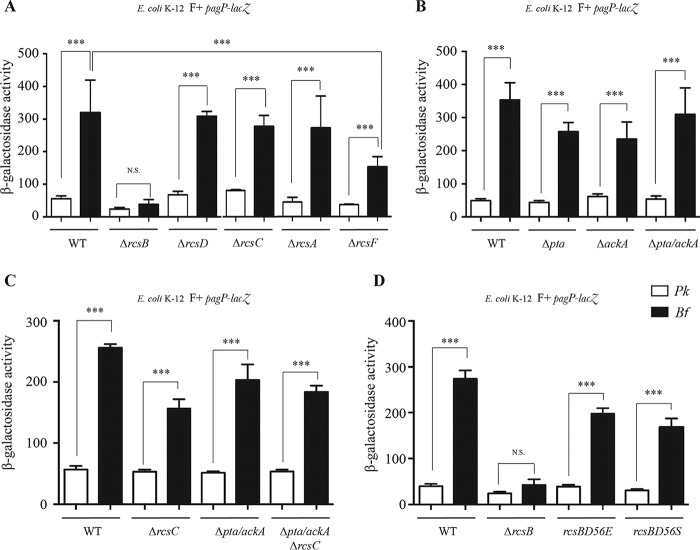
*pagP* transcriptional regulation does not require RcsB phosphorylation. (A) E. coli K-12 MG1655 F^+^ *pagP*-*lacZ* strains, including the wild-type (WT) strain and strains with the *rcsB*, *rcsD*, *rcsC*, *rcsA*, or *rcsF* gene deleted, were grown in planktonic (Pk) cultures and biofilm (Bf) for 48 h, and β-galactosidase activity was measured. (B) E. coli K-12 MG1655 F^+^ *pagP*-*lacZ* strains, including the WT or strains with the *pta* gene or the *ackA* gene or both the *pta* gene and the *ackA* gene deleted, were grown in planktonic (Pk) cultures and biofilm (Bf) for 48 h, and β-galactosidase activity was measured. (C) The E. coli K-12 MG1655 F^+^ *pagP*-*lacZ* WT strain or strains with the *rcsC* or the *pta*/*ackA* genes or both the *rcsC* and *ptA*/*ackA* genes deleted were grown in planktonic (Pk) cultures and biofilm (Bf) for 48 h. β-Galactosidase activity was measured. (D) The E. coli K-12 MG1655 F *pagP*-*lacZ* WT strain and the Δ*rcsB* strain as well as strains *rcsBD56E* and *rcsBD56S* (with a constitutively activated variant and a nonphosphorylatable variant of the *rcsB* gene, respectively), were grown in planktonic (Pk) cultures and biofilm (Bf) for 48 h, and β-galactosidase levels were measured. Statistical significance was assessed using one-way analysis of variance (ANOVA), followed by Bonferroni’s *post hoc* comparison tests (***, *P* < 0.001; N.S., not significant).

The Rcs pathway is composed of several proteins (RcsA, RcsC, RcsD, and RcsF) involved in phosphorylation and dephosphorylation of response regulator RcsB, modulating its ability to bind DNA and regulate target gene expression. To study the contribution of Rcs components to RcsB-dependent induction of *pagP*, we monitored planktonic and biofilm *pagP* expression in several *rcs* mutants and showed that none of the tested *rcs* mutants affected *pagP* expression as much as *rcsB*, suggesting that, in addition to signaling from the outer membrane, *pagP* induction might also be partially independent of the classical Rcs phosphorelay cascade (Fig. 3A).

RcsB is known to be alternatively phosphorylated via an independent pathway that uses acetyl-phosphate (AcP), an intermediate metabolite of the Pta-AckA pathway, as a phosphoryl group donor (22). Although deletion of *ackA* leads to accumulation of AcP in the cell and therefore favors RcsB activation, deletion of both *pta* and *ackA* results in the absence of AcP production. To test whether the acetyl-phosphate level drives *pagP* induction via alternative and Rcs-independent RcsB phosphorylation, we monitored *pagP* induction in a *pta-ackA* mutant background, in which AcP production is abolished (9). We did not observe a significant difference in *pagP* expression in the double *pta*/*ackA* mutant compared to the wild-type strain (Fig. 3B). Moreover, we found that *pagP* expression was still induced in biofilms in a strain combining both *rcsC* and *ackA*/*pta* mutations (Fig. 3C).

These results support the hypothesis that regulation of *pagP* expression does not require RcsB phosphorylation via the Rcs phosphorelay or acetyl phosphate pathway.

Consistently, whereas expression of the *rprA* gene, whose transcription requires the phosphorylated form of RcsB (14, 23), was induced in a strain harboring a constitutively phosphorylated variant of RcsB, RcsB_D56E_, and was decreased in the nonphosphorylatable variant RcsB_D56S_ (Fig. S4), biofilm-specific induction of *pagP* was not impaired in any of the *rcsB* alleles (Fig. 3D).

10.1128/mBio.01415-18.4 FIG S4  *rprA* gene expression requires RcsB phosphorylation. The E. coli K-12 MG1655 F *rprA*-*lacZ* WT strain, the Δ*rcsB* strain, and strains *rcsBD56E* and *rcsBD56S* (harboring a constitutively activated and a nonphosphorylatable variant of the *rcsB* gene, respectively) were grown overnight in planktonic cultures, and β-galactosidase levels were measured. Statistical significance was assessed using one-way analysis of variance (ANOVA), followed by Bonferroni’s *post hoc* comparison tests (***, *P* < 0.001). Download FIG S4, PDF file, 0.1 MB. Copyright © 2018 Szczesny et al. 2018 Szczesny et al. This content is distributed under the terms of the Creative Commons Attribution 4.0 International license.

### Contribution of GadE, SlyA, and H-NS in RcsB-dependent biofilm regulation of *pagP.*

**FIG 4:**
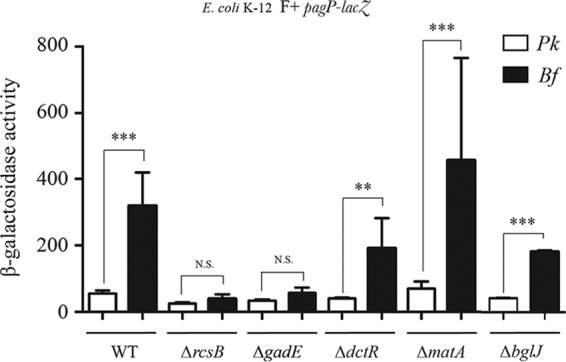
*pagP* biofilm induction requires GadE. E. coli K-12 MG1655 F^+^ *pagP*-*lacZ* strains, wild-type (WT) or with the *rcsB*, *gadE*, *dctR*, *matA*, or *bglJ* gene deleted, were grown in planktonic (Pk) cultures and biofilm (Bf) for 48 h in M63B1 supplemented with 0.4% glucose. Statistical significance was assessed using one-way analysis of variance (ANOVA) followed by Bonferroni’s *post hoc* comparison tests (*, *P* < 0.05; **, *P* < 0.01; ***, *P* < 0.001; N.S., not significant).

**FIG 5:**
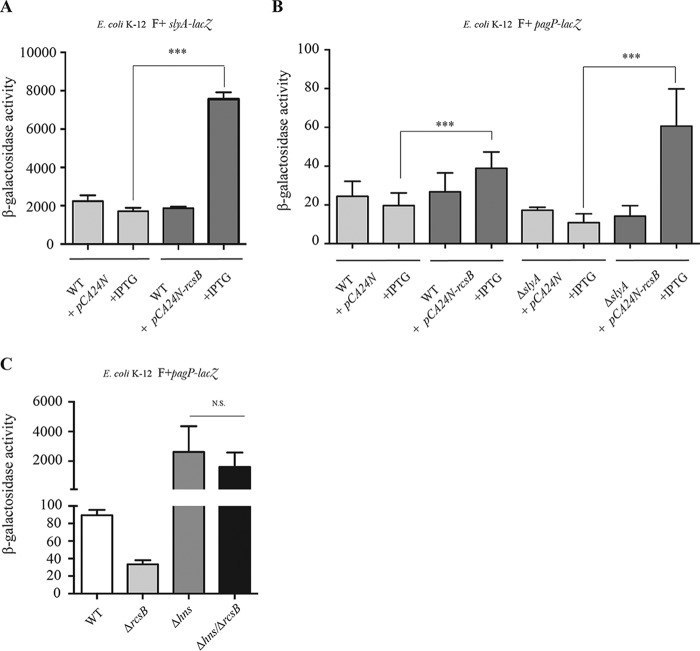
Role of SlyA and H-NS in RcsB-dependent regulation of *pagP*. (A) E. coli K-12 BW25113 F^+^ *slyA*-*lacZ* pCA24N and E. coli K-12 BW25113 F^+^ *slyA*-*lacZ* pCA24N-*lacZ* were grown in planktonic cultures overnight in M63B1 supplemented with 0.4% glucose in the presence or absence of 0.1 mM isopropyl-β-d-thiogalactopyranoside (IPTG), and β-galactosidase levels were measured. (B) E. coli K-12 BW25113 F^+^ pCA24N, E. coli K-12 BW25113 F^+^ pCA24N-*rcsB*, E. coli K-12 BW25113 F^+^ Δ*slyA pagP*-*lacZ* pCA24N, and E. coli K-12 BW25113 F^+^ Δ*slyA pagP*-*lacZ* pCA24N-*rcsB* were grown in planktonic cultures overnight in M63B1 supplemented with 0.4% glucose in the presence or absence of 0.1 mM isopropyl-β-d-thiogalactopyranoside (IPTG), and β-galactosidase levels were measured. (C) E. coli K-12 MG1655 F^+^ *pagP*-*lacZ*, E. coli K-12 MG1655 F^+^ Δ*rcsB pagP*-*lacZ*, E. coli K-12 MG1655 F^+^ Δ*hns pagP*-*lacZ*, and E. coli K-12 MG1655 F^+^ Δ*hns*/Δ*rcsB pagP*-*lacZ* strains were grown under planktonic conditions for 24 h and 48 h, and β-galactosidase activity was measured and statistical significance was assessed using one-way analysis of variance (ANOVA) followed by Bonferroni’s *post hoc* comparison tests (N.S., not significant).

We then tested the potential contribution of several auxiliary transcription regulatory proteins known to interact with nonphosphorylated RcsB, including BglJ, GadE, MatA, and DctR (15, 24–26). We showed that only a mutation in *gadE* abolished biofilm-specific induction of *pagP* (Fig. 4). GadE was previously shown to regulate transcription of the glutamate decarboxylase genes *gadA* and *gadB* in complex with RcsB but independently of RcsB phosphorylation (26). To study the regulatory interplay between H-NS-, SlyA-, and RcsB/GadE-dependent regulation of *pagP* under biofilm conditions, we first tested whether *slyA* transcription is regulated by RcsB. We therefore monitored *slyA* expression in the wild-type strain carrying the pCA24N or pCA24N-*rcsB* inducible plasmid, using a *slyA-lacZ* transcriptional fusion. We showed that overexpression of *rcsB* led to increased expression of *slyA* (Fig. 5A), suggesting that RcsB could positively regulate *slyA* expression, which would in turn positively regulate *pagP*.

However, overexpression of *rcsB* in a Δ*slyA pagP-lacZ* reporter strain still led to increased expression of *pagP* (Fig. 5B). We also tested the possibility that H-NS controls *pagP* expression in an indirect way that involves repression of the positive regulator *rcsB*. However, deletion of *rcsB* in an *hns*-deficient strain, in which *pagP* expression is derepressed, did not restore the wild-type level of *pagP* expression (Fig. 5C). These results therefore suggest that, while RcsB acts upstream of SlyA, other SlyA-independent inputs can contribute to alleviate H-NS-mediated repression of *pagP* (see model in Fig. 7).

### Increased ionic strength triggers RcsB- and biofilm-dependent *pagP* expression.

**FIG 6:**
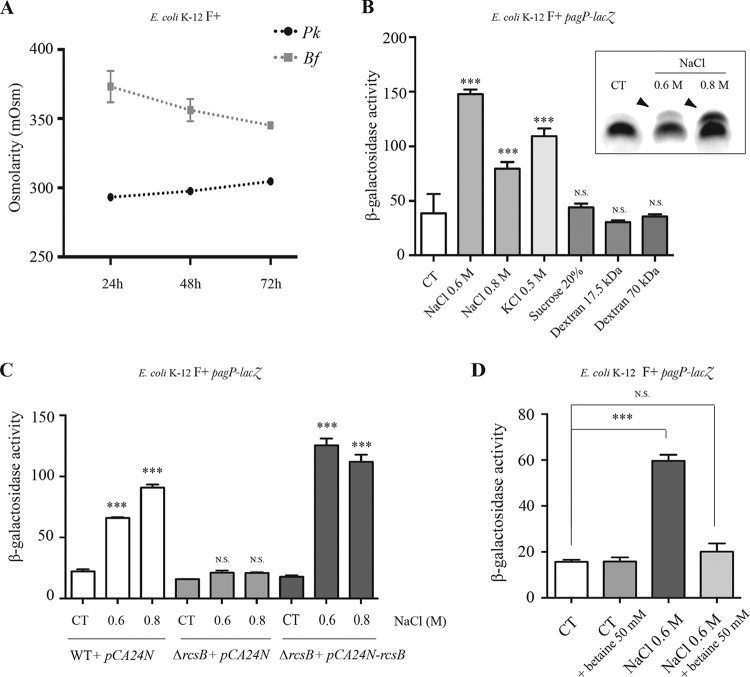
*pagP* transcriptional induction is osmotically regulated. (A) E. coli K-12 MG1655 F^+^ was grown under planktonic (Pk) or biofilm (Bf) conditions for 24, 48, and 72 h, and the osmolarity of the culture medium or biofilm supernanatant was measured using an osmometer. (B) E. coli K-12 MG1655 F^+^ *pagP*-*lacZ* was grown in planktonic cultures to an OD of 0.4 of 0.5 and exposed for 1 h to NaCl (0.6 or 0.8 M), KCl (0.5 M), sucrose (20%), and dextran (17.5 kDa or 70 kDa at 10%), and β-galactosidase activity was measured. Inset: analysis of LPS extracted from control and NaCl-exposed (0.6 M or 0.8 M) planktonic bacteria by Tricine SDS-PAGE. Arrowheads indicate a modified LPS band. (C) E. coli K-12 BW25113 F^+^ *pagP*-*lacZ* pCA24N, E. coli K-12 BW25113 F^+^ Δ*rcsB pagP*-*lacZ* pCA24N, and E. coli K-12 BW25113 F^+^ Δ*rcsB pagP*-*lacZ* pCA24N-*rcsB* were grown in planktonic cultures in M63B1 supplemented with 0.4% glucose and 0.1 mM isopropyl-β-d-thiogalactopyranoside (IPTG) to an OD of 0.4 to 0.5. NaCl (0.6 or 0.8 M) was added to the cultures, and β-galactosidase activity was measured after 1 h of incubation. (D) E. coli K-12 MG1655 F^+^ *pagP*-*lacZ* was grown in planktonic cultures to an OD of 0.4 to 0.5 and exposed to 0.6 M of NaCl in the presence or absence of betaine (an osmoprotective osmolyte). β-Galactosidase activity was measured after 1 h of incubation. Statistical significance was assessed against the corresponding untreated control (CT) using one-way analysis of variance (ANOVA) followed by Bonferroni’s *post hoc* comparison tests (***, *P* < 0.001; N.S., not significant).

The implication of stress response regulators RcsB and GadE in regulation of *pagP* expression suggested that biofilm-associated induction of *pagP* could be triggered in response to physicochemical stress conditions created locally within the biofilm environment (27). The Rcs envelope stress system is known to respond to several physicochemical cues, including osmotic stress and exposure to highly ionic environments (20). To test whether RcsB/GadE-dependent expression of *pagP* is activated in response to osmotic stress in biofilm, we measured the osmolarity of planktonic and biofilm supernatants of E. coli after 24, 48, and 72 h of growth and showed that the osmolarity was consistently higher in biofilm supernatants than in planktonic samples (Fig. 6A). We further studied the contribution of osmotic stress to *pagP* gene expression by briefly exposing exponentially growing bacteria to salts such as NaCl or KCl and to polymers (sucrose or dextran) commonly used for studying bacterial response to osmotic stress, and we then monitored *pagP* expression. A significant increase in *pagP* gene expression was observed only when bacteria were exposed to NaCl or KCl (0.5 to 0.8 M) (Fig. 6B), and we found that this induction was dependent on *rcsB* (Fig. 6C). Consistently, monitoring the lipid A palmitoylation profile on LPS gels in the presence or absence of NaCl showed that the additional band corresponding to palmitoylated lipid A was observed when bacteria were exposed to NaCl (0.6 and 0.8 M) (Fig. 6B) and that the impact of NaCl was strongly reduced in the *rcsB* mutant strain (Fig. S5). Moreover, NaCl-dependent induction of *pagP* was strongly reduced in planktonic bacteria upon addition of the osmoprotectant osmolyte betaine (Fig. 6D), suggesting that ionic stress triggers *pagP* expression.

10.1128/mBio.01415-18.5 FIG S5  Role of RcsB in NaCl-dependent induction of lipid A palmitoylation. Tricine SDS-PAGE/periodate-silver staining analysis of LPS extracted from control and NaCl-exposed (0.6 M or 0.8 M) planktonic E. coli K-12 MG1655 F^+^ WT and Δ*rcsB* strains was performed. The arrow indicates a modified LPS band. Download FIG S5, PDF file, 0.2 MB. Copyright © 2018 Szczesny et al. 2018 Szczesny et al. This content is distributed under the terms of the Creative Commons Attribution 4.0 International license.

In E. coli, sensory proteins such as the KdpD sensor of the KdpDE two-component system can detect changes in osmolarity (28). Moreover, mechanosensitive channels such as that corresponding to the MscL membrane protein also participate in regulation of turgor pressure by modulating ion fluxes (29). To test whether changes in ion fluxes could activate RcsB-GadE-dependent regulation of lipid A palmitoylation, we monitored *pagP* induction in biofilms formed by *trkA*, *kdpD*, *mscL*, or *mscS* mutants but could not detect any impact on *pagP* transcriptional induction in biofilm (Fig. S6).

10.1128/mBio.01415-18.6 FIG S6  Impact of deletion of ion transporters on *pagP* gene expression. E. coli K-12 MG1655 F^+^ *pagP*-*lacZ* strains, WT or with the *trkA*, *kdpD*, *mscL*, or *mscS* gene deleted, were grown in planktonic (Pk) and biofilm (Bf) for 48 h. β-Galactosidase activity was measured. Statistical significance was assessed using one-way analysis of variance (ANOVA) followed by Bonferroni’s *post hoc* comparison tests (***, *P* < 0.001). Download FIG S6, PDF file, 0.1 MB. Copyright © 2018 Szczesny et al. 2018 Szczesny et al. This content is distributed under the terms of the Creative Commons Attribution 4.0 International license.

The GadE stress regulator is involved in maintenance of pH homeostasis and is induced under low-pH conditions (30). We therefore measured the pH of planktonic and biofilm supernatants of E. coli after 24, 48, and 72 h of growth to test whether expression of *pagP* is activated in response to pH changes and showed that the pH of biofilm supernatants is acidified compared to planktonic samples (Fig. S7A). However, growing planktonic bacteria at different pH levels had only a limited impact on *pagP* gene expression, indicating that *pagP* transcription is not induced by acid stress (Fig. S7B). Moreover, whereas *gadE* is required to induce *pagP* expression in biofilms, NaCl-dependent induction of *pagP* in planktonic bacteria was not abolished in a Δ*gadE* mutant (Fig. S8), suggesting that exposure to NaCl partially mimics conditions created in the biofilm environment.

10.1128/mBio.01415-18.7 FIG S7  Effect of pH variation on *pagP* expression. (A) E. coli K-12 MG1655 F^+^ was grown under planktonic (Pk) or biofilm (Bf) conditions for 24, 48, and 72 h, and the pH of the culture medium or biofilm supernanatant was measured using pH indicator strips. (B) E. coli K-12 MG1655 F^+^ *pagP-lacZ* was grown overnight in planktonic cultures in M63B1 supplemented with 0.4% glucose adjusted at different pH levels, and β-galactosidase activity was measured. Statistical significance (compared to the control at pH 7) was assessed using one-way analysis of variance (ANOVA) followed by Bonferroni’s *post hoc* comparison tests (*, *P* < 0.05; **, *P* < 0.01; NS, not significant). Download FIG S7, PDF file, 0.1 MB. Copyright © 2018 Szczesny et al. 2018 Szczesny et al. This content is distributed under the terms of the Creative Commons Attribution 4.0 International license.

10.1128/mBio.01415-18.8 FIG S8  NaCl-dependent induction of *pagP* in planktonic cultures does not require GadE. E. coli K-12 MG1655 F^+^ *pagP-lacZ*, E. coli K-12 MG1655 F^+^ Δ*rcsB pagP-lacZ*, and E. coli K-12 MG1655 F^+^ Δ*gadE pagP-lacZ* strains were grown in planktonic cultures to an OD of 0.5 and exposed for 1 h to NaCl (0.6 M or 0.8 M). β-Galactosidase activity was measured, and statistical significance was assessed against the corresponding control without NaCl (CT) using one-way analysis of variance (ANOVA) followed by Bonferroni’s *post hoc* comparison tests (***, *P* < 0.001; NS, not significant). Download FIG S8, PDF file, 0.1 MB. Copyright © 2018 Szczesny et al. 2018 Szczesny et al. This content is distributed under the terms of the Creative Commons Attribution 4.0 International license.

These results demonstrate that increased ionic stress can trigger *pagP* expression in a RcsB-dependent and GadE-independent manner and suggest that increased ionic stress associated with increased osmolarity could be part of the signal activating RcsB and inducing *pagP* expression and lipid A palmitoylation in E. coli biofilms.

## DISCUSSION

LPS is a major component of the Gram-negative bacterial outer membrane and possesses a highly dynamic structure responding to environmental changes (31). We previously showed that formation of Gram-negative bacterial biofilms is associated with transient palmitoylation of lipid A mediated by palmitoyl transferase enzyme PagP, contributing to protection of biofilm bacteria from the host immune system (7). Here we investigated mechanisms underlying induction of this biofilm-associated LPS modification.

Increased lipid A palmitoylation is a general feature of Gram-negative bacterial biofilms which, in E. coli, can result either from transcriptional induction of *pagP* or from posttranslational activation of the PagP enzyme in response to perturbations of outer membrane lipid asymmetry (12). In prolonged planktonic cultures, absence of transcriptional induction of *pagP* while palmitoylation occurs suggests that, under these growth conditions, increased lipid A modification could result solely from posttranslational activation of PagP. Stress created under these extreme stationary-phase conditions could alter the integrity of the outer membrane, for instance, by causing phospholipid substrate migration to the outer leaflet, where they can activate the basal level of resident PagP molecules (32, 33). Given the well-established stationary-phase character of the biofilm environment (34), it is likely that a significant proportion of the lipid A palmitoylation observed in biofilm bacteria also originates from posttranslational activation of PagP.

**FIG 7:**
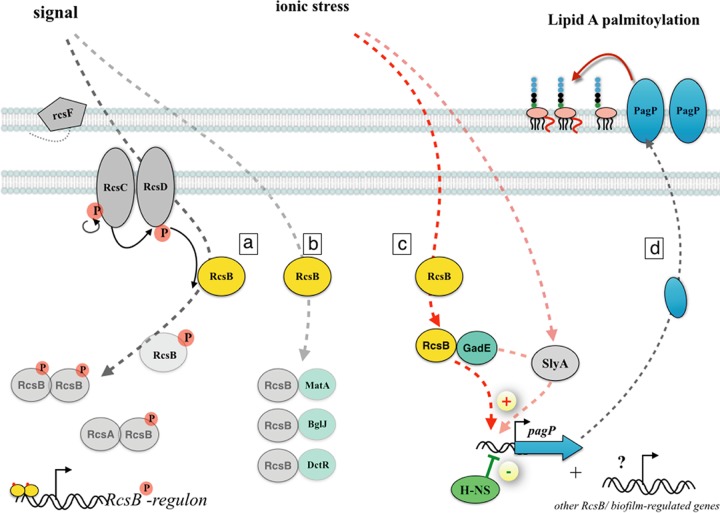
Proposed model for biofilm transcriptional regulation of *pagP* expression. The Rcs system is a two-component signal transduction system that responds to various environmental signals. (Pathway a) Proteins (RcsA, RcsC, RcsD, and RcsF) involved in phosphorylation and dephosphorylation of response regulator RcsB are not required for *pagP* biofilm induction. (Pathway b) RcsB regulation is also independent of MatA, BglJ, and DctR, known to interact with the nonphosphorylated form of RcsB. (Pathway c) In response to ionic stress, RcsB and GadE could form a complex positively regulating *pagP* expression in a manner at least partially independent of the anti-H-NS factor SlyA. (Pathway d) This countersilencing mechanism leads to *pagP* biofilm induction, as well as to an increased level of lipid A palmitoylation, and could potentially lead to expression of additional biofilm-regulated genes.

In addition to potential posttranslational activation of PagP, we showed that the biofilm environment triggers RcsB-dependent transcriptional induction of *pagP*, also contributing to the global increase in lipid A palmitoylation. This effect was not detected in our previous study (7), probably due to the parallel confounding increase in posttranslational activation of PagP under these conditions. The Rcs phosphorelay system regulates colanic acid synthesis, cell division, growth on surfaces, and motility, all important functions involved in biofilm formation and bacterial physiological adaptation to the biofilm lifestyle (21, 35). Several major and accessory components (RcsF, RcsC, RcsD, and RcsA) are involved in RcsB phosphorylation and subsequent regulation of a subset of Rcs-regulated genes (36). Here we show that RcsB regulates *pagP* expression independently of major contributions of other Rcs components and of its phosphorylation. Consistently, we discovered that the auxiliary transcriptional regulator GadE, one of the 4 transcriptional regulators known to interact with the nonphosphorylated form of RcsB (MatA, BglJ, DtcR, and GadE) (24, 26), contributes to RcsB-dependent regulation of *pagP* induction. GadE is expressed under low-pH conditions (30, 37), whereas RcsB can be activated by several stress conditions, including membrane perturbations (38, 39), osmotic stress (20), and growth on solid surfaces (40). Since the RcsB-BglJ complex specifically regulates at least 10 genes, 8 of which correspond to unknown or predicted proteins (41), this suggests that, in addition to *pagP*, other genes regulated by the RcsB-GadE complex remain to be discovered, and their discovery could lead to identification of novel biofilm properties. Although the RcsB-GadE complex was reported to regulate only *gadA*/*BC* genes (26), it is worth noting that the glutamate decarboxylase genes *gadA* and *gadB* are, like *pagP*, under the negative control of H-NS (Fig. 7) and were shown to be induced in biofilm (34, 42, 43).

Our results suggest that, in addition to *pagP*, conditions prevailing within biofilm could specifically trigger the expression of other RcsB-regulated and H-NS-regulated genes. Consistently, the expression of RcsB-regulated genes *cpsB* and *rprA*, or of RcsB/GadE-regulated genes *gadA* and *gadB*, showed significant induction under biofilm conditions (see Fig. S9 in the supplemental material) (44, 45). Hence, RcsB could play a central role in H-NS antisilencing and could enable the expression of *rcs* genes regulated both through activation of the phosphorelay cascade and through activation of RcsB complexes formed with auxiliary regulators. Consistently, among genes positively regulated by the RcsB-BglJ complex, *leuO* is an anti-H-NS factor (46, 47) that could, when expressed, contribute to global antagonism of H-NS-mediated repression. However, it has been shown that all RcsB auxiliary regulators, like some of their targets, are repressed by H-NS (48–51), raising the question of how their expression can be activated. Since H-NS preferentially binds to curved and A-T-rich DNA regions (52, 53), alteration of DNA structure under environmental conditions reducing the degree of DNA curvature, such as high temperature or increased osmolarity (54), could lead to H-NS destabilization and derepression of H-NS-repressed genes.

10.1128/mBio.01415-18.9 FIG S9  *cpsB*, *rprA*, and *gadA*/*B* are induced under biofilm conditions. E. coli K-12 MG1655 F^+^ *cpsB-lacZ*, E. coli K-12 MG1655 F^+^ *rprA-lacZ*, E. coli K-12 MG1655 F^+^ *gadA-lacZ*, and E. coli K-12 MG1655 F^+^ *gadB-lacZ* strains were grown under planktonic (Pk) and biofilm (Bf) conditions for 48 h, and β-galactosidase activity was measured. Statistical significance was assessed using one-way analysis of variance (ANOVA) followed by Bonferroni’s *post hoc* comparison tests (*, *P* < 0.05; **, *P* < 0.01; ***, *P* < 0.001). Download FIG S9, PDF file, 0.1 MB. Copyright © 2018 Szczesny et al. 2018 Szczesny et al. This content is distributed under the terms of the Creative Commons Attribution 4.0 International license.

The Rcs system was previously described as being activated by various cues such as LPS or peptidoglycan defects, temperature, or osmotic shock (16). We showed that RcsB-dependent *pagP* expression and increased lipid A palmitoylation can be induced by ionic solutes but not by nonionic compounds such as sucrose or dextran. The mechanisms behind this specificity could involve an osmotic stress response triggered to minimize loss of water and maintain positive turgor pressure (55). In bacteria subjected to salt stress, one key mechanism of bacterial osmoadaptation lies in rapid K^+^ uptake through different K^+^ sensory and transport systems (56). However, deletion of major components of the E. coli potassium transporters or mechanosensitive channels had no impact on *pagP* transcriptional induction in biofilm. Further investigations will determine the contribution of additional ion transporters to biofilm-associated *pagP* induction.

Consistently with NaCl-dependent and KCl-dependent induction of *pagP*, we detected higher osmolarity in the biofilm environment, suggesting that increased osmolarity could be the signal triggering RcsB-dependent induction within biofilms. In Dickeya dadantii, the level of osmoregulated periplasmic glucans (OPGs), a periplasmic component of the cell envelope, was shown to modulate the activity of the Rcs system. While accumulation of OPGs in the periplasm inhibits the Rcs pathway under conditions of low osmolarity, in biofilms, which are characterized by higher osmolarity, absence of OPGs could serve as a signal activating the Rcs pathway and leading to increased expression of *pagP* (57). While the origin of this increased osmolarity is not known, it might be explained by accumulation of metabolic wastes, cell lysis, or production of matrix components that characterize the biofilm mode of growth. Increased osmolarity could therefore be a general feature regulating other biofilm-specific responses beyond lipid A modifications. Interestingly, several examples of membrane modifications in response to osmotic stress have been described, including modification of the LPS structure in Rhizobium meliloti (58) and the increase in the amount of saturated fatty acids in Pseudomonas putida colonies, contributing to increased membrane rigidity (59). While PagP-dependent palmitoylation was also shown to contribute to membrane rigidity (60), we speculate that this phenotype is part of a more global mechanism for adaptation to life under conditions of increased osmolarity and contributes to maintaining cell homeostasis by limiting loss of water.

In Fig. 7 we present a model for the *pagP* transcriptional regulation in biofilm described in this study. We propose that the biofilm environment, characterized by increased osmolarity, triggers activation of RcsB and its interaction with the auxiliary regulator GadE. Formation of a RcsB-GadE complex, occurring independently of RcsB phosphorylation, leads to positive regulation of *pagP* expression. Our results further suggest that this new regulatory pathway could be at least partially independent of the previously described anti-H-NS SlyA factor.

The analyses presented in this study were performed on a biofilm population. The observed increased lipid A palmitoylation could be interpreted as a general feature of all biofilm cells. However, biofilm environments are highly heterogeneous, and, depending on their localization within the biofilm, bacteria encounter different stress conditions (27). It is therefore possible that the level of lipid A palmitoylation that we observe in biofilm reflects an average level between those of highly palmitoylated and nonpalmitoylated cells which, in addition, could result either from increased *pagP* expression or from activation of the basal level of the dormant enzyme. The resulting heterogeneity among biofilm cells could represent an adaptive advantage ensuring population survival in hostile environments. Consistently, our previous *in vivo* analyses showed that lipid A palmitoylation increased biofilm bacterial survival by reducing the host inflammatory response (7). However, while the presence of highly palmitoylated lipid A at the surface of the biofilm could represent a defensive barrier preventing biofilm eradication by the host immune system, our results suggest instead that the conditions required for *pagP* induction are more closely correlated with deeper biofilm layers in which accumulation of metabolites and other wastes could increase external osmolarity. Further localization studies with oxygen-insensitive reporters or imaging mass spectrometry will help clarify the correlation between induction of *pagP* and the physicochemical cues present in the biofilm environment.

## MATERIALS AND METHODS

### Bacterial strains and growth conditions.

Bacterial strains and plasmids used in this study are described in Table S1 in the supplemental material. Antibiotics (concentrations) were used as follows: kanamycin (50 µg/ml); chloramphenicol (25 µg/ml); ampicillin (100 µg/ml); zeocin (50 µg/ml); and tetracycline (7.5 µg/ml). When specified, isopropyl-β-d-thiogalactopyranoside (IPTG) was used to induce gene expression at the following concentrations: for planktonic cultures, 0.1 mM; for biofilms, 0.05 mM.

10.1128/mBio.01415-18.10 TABLE S1  Strains and plasmids used in this study. Download TABLE S1, PDF file, 0.1 MB. Copyright © 2018 Szczesny et al. 2018 Szczesny et al. This content is distributed under the terms of the Creative Commons Attribution 4.0 International license.

### Biofilm growth in continuous-flow microfermentors.

Biofilm formation on an internal glass slide stand in continuous-flow microfermentors was performed as previously described (18). Inoculation was performed by dipping the glass spatula for 5 min in a culture adjusted to an optical density at 600 nm (OD_600_) of 2 from overnight bacterial cultures grown in M63B1 minimal medium supplemented with 0.4% glucose and the appropriate antibiotics. The spatula was then reintroduced into the microfermentor, and biofilm culture was performed at 37°C in M63B1 with glucose. Biofilm biomass produced at different time points was rapidly resuspended in 10 ml of fresh M63B1 medium (without glucose), and biofilm bacteria were used for LPS analysis of measures of β-galactosidase activity.

### Planktonic growth.

Planktonic cultures were also grown in M63B1 medium supplemented with 0.4% glucose at 37°C.

### Strain construction.

The E. coli deletion mutants used in these studies either originated from E. coli K-12 BW25113 wild-type and isogenic mutant strains from the Keio collection (61) or were derived by λ-red linear DNA gene inactivation using pKOBEG plasmids or derivates (62, 63). To construct *pagP*-*lacZ* derivatives, mutations were transferred by P1vir phage transduction to BW25113 *pagP*-*lacZ*, BW25113 F^+^ *pagP*-*lacZ*, MG1655 *pagP*-*lacZ*, or MG1655 F^+^ *pagP*-*lacZ* strains. When required, kanamycin resistance markers flanked by two FLP recombination target (FRT) sites were removed using Flp recombinase (64).

### LPS analysis by Tricine SDS-PAGE.

A total of 10^8^ bacteria were pelleted and resuspended in 100 µl of lysing buffer (Bio-Rad) containing 1% SDS, 20% glycerol, 100 mM Tris (pH 6.8), and Coomassie blue G-250. Lysates were heated at 100°C for 10 min; proteinase K was then added at 1 mg/ml, and the reaction mixture was incubated at 37°C for 1 h. These samples (4 µl) were electrophoresed in a Tricine SDS-PAGE system, which improves resolution of the low-molecular-weight LPS band, using 4% stacking gel and 20% separating gel (65). LPS was then visualized by a periodate-silver staining method adapted from a previous study (66). Gels were immersed in fixing solution (30% ethanol, 10% acetic acid) for 1 h, washed in water three times for 5 min each time, oxidized in 0.7% periodic acid for 10 min, and washed in water three times for 5 min each time. Gels were then immersed for 1 min in 0.02% thiosulfate pentahydrate, rinsed quickly in water, and stained in 25 mM silver nitrate for 10 min. After a 15 s wash in water, gels were developed in 3.5% potassium carbonate–0.01% formaldehyde. Development was stopped for 30 min in 4% Tris base–2% acetic acid.

### Direct lipid A isolation from bacterial cells.

Lipid A was isolated directly by hydrolysis of bacterial cells as described previously (67, 68). Briefly, 5 mg of lyophilized bacterial cells was suspended in 100 µl of a mixture of isobutyric acid–1 M ammonium hydroxide (5:3 [vol/vol]) and kept for 1.5 h at 100°C in a screw-cap test tube in a Thermomixer system. The suspension was cooled in ice water and centrifuged (2,000 × *g*, 5 min). The recovered supernatant was diluted with 2 volumes of water and lyophilized. The sample was then washed once with 100 µl of methanol (by centrifugation at 2,000 × *g* for 5 min). Finally, lipid A was extracted from the pellet in 50 µl of a mixture of chloroform, methanol, and water (3:1.5:0.25 [vol/vol/vol]).

### MALDI-TOF MS analysis.

LPS samples were dispersed in water at 1 µg/µl. Lipid A extracts in chloroform-methanol-water were used directly in this mixture of solvents. In both cases, a volume of a few microliters of sample solution was desalted with a few grains of ion exchange resin (Dowex 50W-8 [H^+^]). Aliquots of 0.5 to 1 µl of the solution were deposited on the target, and the spot was then overlaid with matrix solution and left to dry. Dihydroxybenzoic acid (DHB) (Sigma-Aldrich) or 5-chloro-2-mercaptobenzothiazole (CMBT) was used as the matrix. It was dissolved at 10 mg/ml in 0.1 M citric acid solution in the same solvents as were used for the analytes (69). Different analyte/matrix ratios (1:2, 1:1, and 2:1 [vol/vol]) were tested to obtain the best spectra. Negative- and positive-ion mass spectra were recorded on a PerSeptive Voyager-DE short tandem repeat (STR) time of flight mass spectrometer (Applied Biosystems) in the linear mode with delayed extraction. The ion-accelerating voltage was set at −20 kV, and the extraction delay time was adjusted to obtain the best resolution and signal-to-noise ratio. LPS analyses were performed by LPS-BioSciences (IGM, Orsay, France).

### β-Galactosidase activity assay.

To determine the β-galactosidase enzyme activity of strains carrying a transcriptional *lacZ* fusion, bacteria were grown in M63B1 minimal medium with glucose at 37°C for the time indicated for each experiment. The enzyme activity was assayed at least in triplicate for each strain under each condition as previously described (70), and the results are expressed in Miller units.

### Screening for conditions impacting lipid A palmitoylation and *pagP* expression.

To evaluate the roles of different physicochemical stresses in *pagP* expression and lipid A palmitoylation, we proceeded as follows.

### (i) Test of osmotic agents.

Planktonic cultures in M63B1 minimal medium supplemented with 0.4% glucose and the appropriate antibiotics were started at an optical density at 600 nm (OD_600_) of 0.05 from overnight precultures performed in LB. Bacteria were incubated at 37°C until they reached an optical density at 600 nm (OD_600_) approaching 0.5. The tested chemical was then added to the cultures, and β-galactosidase activity was measured after 1 h if incubation. NaCl, KCl, sucrose, and dextran were used to induce osmotic stress. The listed compounds (concentrations) were used as follows: NaCl (0.6 M and 0.8 M); KCl (0.5 M); 17.5-kDa and 70-kDa dextran (10%); and sucrose (20%).

### (ii) Growth at different pH levels.

Starting from a preculture performed in LB, bacteria were grown overnight in M63B1 minimal medium supplemented with 0.4% glucose and adjusted at different pH levels (pH 5.2, 6, 6.5, 7, and 7.4). Medium pH was buffered using 3-morpholinopropane-1-sulfonic acid (MOPS) or 2-(N-morpholino)ethane sulfonic acid (MES) at 100 mM. β-Galactosidase activity was measured.

### Osmolarity and pH measurements.

To determine the osmolarity level and the pH in planktonic and biofilm cultures, we proceeded as follows.

### (i) Sample preparations.

For biofilm measurements, the biofilm biomass was recovered from the glass spatula using a cell scraper to minimize dilution of the biofilm supernatant. The biofilm sample was centrifuged and filtered to eliminate residual bacteria. For planktonic cultures, an aliquot of the bacterial suspension was centrifuged and filtered.

Samples were recovered from at least 3 independent biofilm and planktonic cultures and 3 different time points (24 h, 48 h, and 72 h).

### (ii) Measures.

Osmolarity measurements were performed on 100-µl samples using a freezing-point osmometer and repeated 3 times. pH was assessed using pH strips (Sigma P-4536) with a unit resolution of 0.5 pH.

### Statistical analysis.

Statistical analyses were performed using one-way analysis of variance (ANOVA) and the Bonferroni multiple-comparison test with Prism 5.0 for Mac OS X (GraphPad Software, Inc.). Each experiment was performed at least 3 times. Statistical significance is indicated as follows: *, *P* = <0.05; **, *P* = <0.01; ***, *P* = <0.001.
