# Supplementary material for: Increased Osmolarity in Biofilm Triggers RcsB-Dependent Lipid A Palmitoylation in Escherichia coli
Source: mBio. 2018 Aug 21;9(4):e01415-18. doi: 10.1128/mBio.01415-18 (PMC6106083; doi:10.1128/mBio.01415-18)
Supplement: FIG S1 [file mbo004184028sf1.pdf]

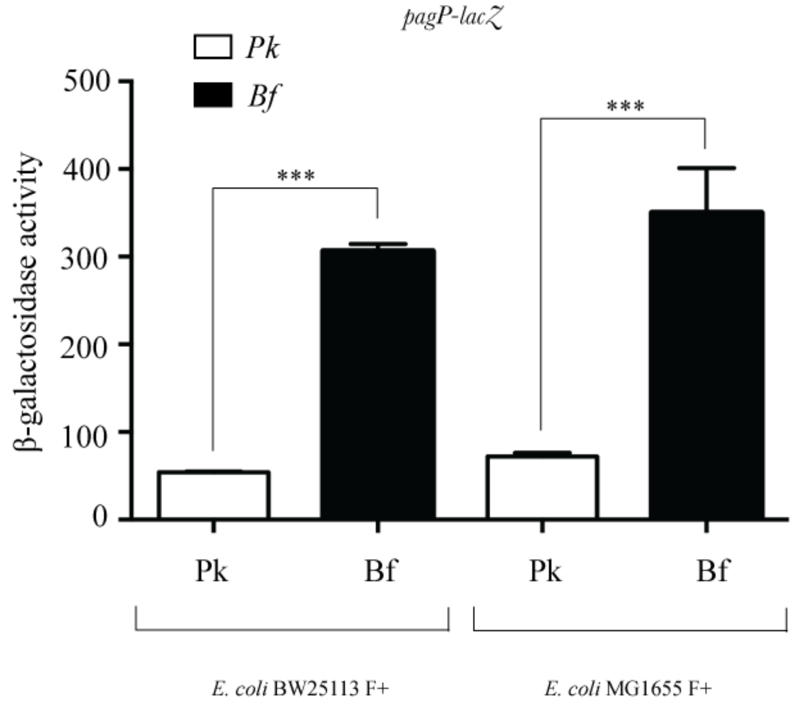

Supplementary Figure S1. ***pagP* biofilm induction in *E. coli* K-12 BW25113 and MG1655**.

Comparison of *pagP* expression in *E. coli* K-12 BW25113 F+ *pagP-lacZ* and *E. coli* K-12 MG1655 F+ *pagP-lacZ* grown under planktonic (Pk) or biofilm (Bf) conditions. Due to the presence of the biofilm-promoting F conjugative plasmid, the time of growth was reduced to 48 h. β-galactosidase activity was measured after 1 h of incubation. β-galactosidase activity was measured. Statistical significance was assessed using one-way analysis of variance (ANOVA), followed by *Bonferroni's* post-hoc comparisons (*** $p < 0.001$).
