# Supplementary material for: Increased Osmolarity in Biofilm Triggers RcsB-Dependent Lipid A Palmitoylation in Escherichia coli
Source: mBio. 2018 Aug 21;9(4):e01415-18. doi: 10.1128/mBio.01415-18 (PMC6106083; doi:10.1128/mBio.01415-18)
Supplement: FIG S2 [file mbo004184028sf2.pdf]

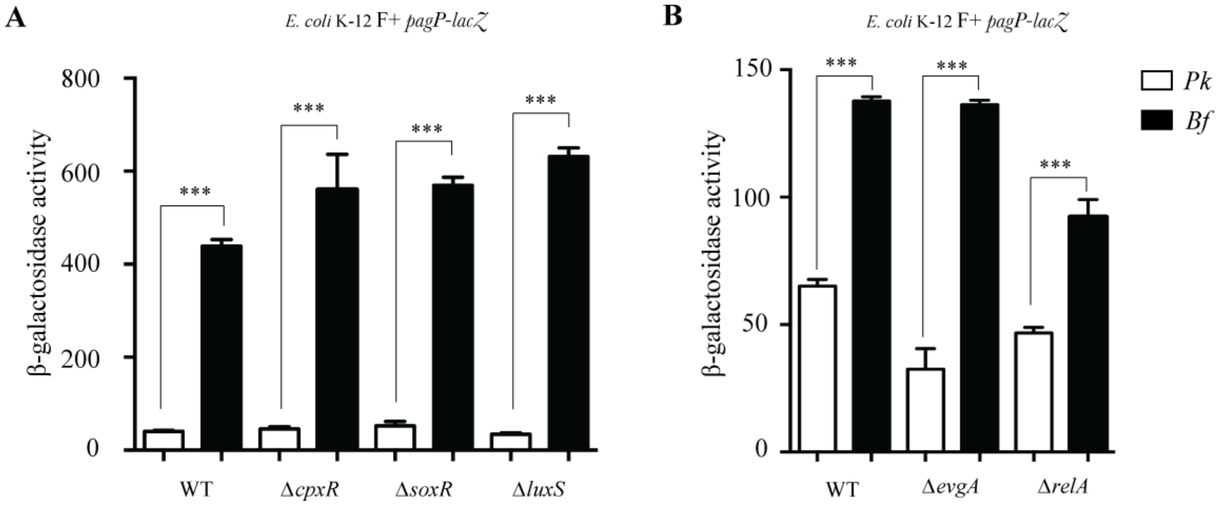

Supplementary Figure S2. **Impact of deletion of regulators on *pagP* biofilm induction.** *E. coli* K-12 BW25113 F+ *pagP-lacZ* strains, WT or with *cpxR, soxR, luxS* **(A)** or *evgA, relA* **(B)** gene deleted, were grown in planktonic conditions (Pk) and biofilm (Bf) for 48 h. β-galactosidase activity was measured. Statistical significance was assessed using one-way analysis of variance (ANOVA) followed by *Bonferroni's* post-hoc comparison *tests* (*** $p < 0.001$).
