# Supplementary material for: Increased Osmolarity in Biofilm Triggers RcsB-Dependent Lipid A Palmitoylation in Escherichia coli
Source: mBio. 2018 Aug 21;9(4):e01415-18. doi: 10.1128/mBio.01415-18 (PMC6106083; doi:10.1128/mBio.01415-18)
Supplement: FIG S3 [file mbo004184028sf3.pdf]

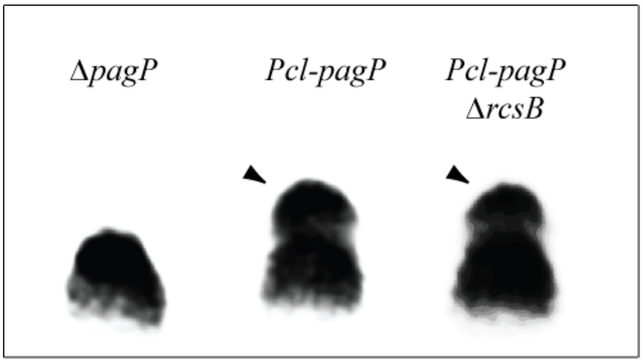

Supplementary Figure S3. **Deletion of *rcsB* does not impact PagP at a post-translational level.**

Tricine SDS-PAGE/periodate-silver staining analysis of LPS extracted from planktonic *E. coli* K-12 MG1655 F+ *ΔpagP,* MG1655 F+ *PcL-pagP* and MG1655 F+ *PcL-pagP* Δ*rcsB* LPS were analyzed from exponentially growing cells in M63B1 0.4% glucose; arrows indicate a modified LPS band
