# Supplementary material for: Increased Osmolarity in Biofilm Triggers RcsB-Dependent Lipid A Palmitoylation in Escherichia coli
Source: mBio. 2018 Aug 21;9(4):e01415-18. doi: 10.1128/mBio.01415-18 (PMC6106083; doi:10.1128/mBio.01415-18)
Supplement: FIG S4 [file mbo004184028sf4.pdf]

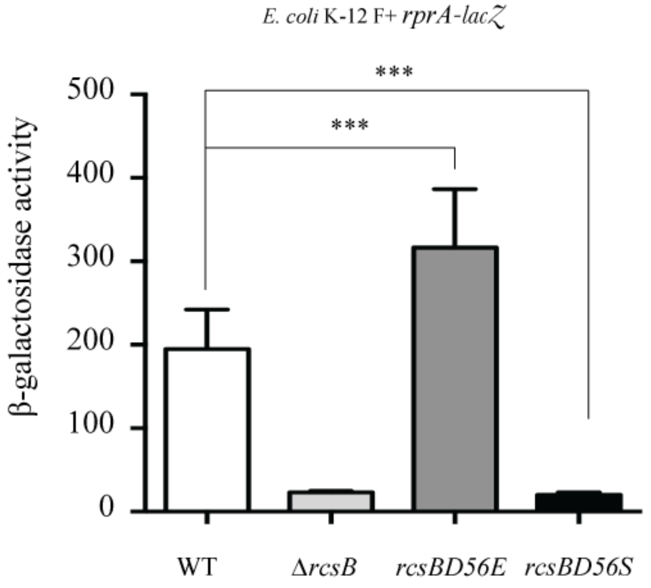

Supplementary Figure S4: ***rprA*** **gene expression requires RcsB phosphorylation.**

E. coli K-12 MG1655 F *rprA-lacZ* strains WT, Δ*rcsB, rcsBD56E* and *rcsBD56S* (a constitutively activated and a non-phosphorylable variant of the *rcsB* gene, respectively) were grown overnight in planktonic cultures and β-galactosidase was measured. Statistical significance was assessed using one-way analysis of variance (ANOVA), followed by *Bonferroni's* post-hoc comparison *tests* (*** $p < 0.001$).
