# Supplementary material for: Increased Osmolarity in Biofilm Triggers RcsB-Dependent Lipid A Palmitoylation in Escherichia coli
Source: mBio. 2018 Aug 21;9(4):e01415-18. doi: 10.1128/mBio.01415-18 (PMC6106083; doi:10.1128/mBio.01415-18)
Supplement: FIG S5 [file mbo004184028sf5.pdf]

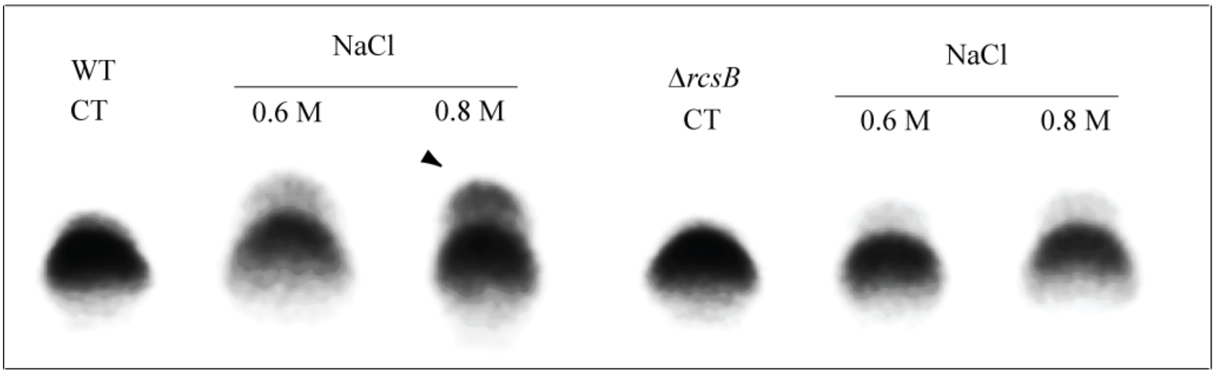

Supplementary Figure S5: Role of RcsB in Nacl-dependent induction of lipid A palmitoylation.

Tricine SDS-PAGE/periodate-silver staining analysis of LPS extracted from control and NaCl-exposed (0.6M or 0.8 M) planktonic *E. coli* K-12 MG1655 F+ WT and Δ*rcsB*. Arrow indicate a modified LPS band.
