# Supplementary material for: Increased Osmolarity in Biofilm Triggers RcsB-Dependent Lipid A Palmitoylation in Escherichia coli
Source: mBio. 2018 Aug 21;9(4):e01415-18. doi: 10.1128/mBio.01415-18 (PMC6106083; doi:10.1128/mBio.01415-18)
Supplement: FIG S6 [file mbo004184028sf6.pdf]

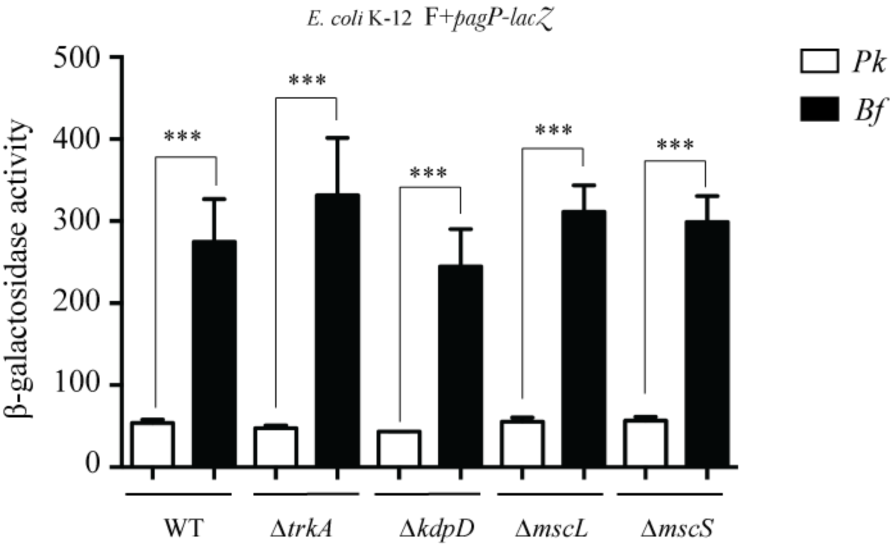

Supplementary Figure S6. **Impact of deletion of ion transporters on *pagP* gene expression.**

E. coli K-12 MG1655 F+ *pagP-lacZ* strains, WT or with the *trkA, kdpD, mscL* or *mscS* gene deleted, were grown in planktonic (Pk) and biofilm (Bf) for 48 h. β-galactosidase activity was measured. Statistical significance was assessed using one-way analysis of variance (ANOVA) followed by *Bonferroni's* post-hoc comparisons *tests* (*** $p < 0.001$)
