# Supplementary material for: Increased Osmolarity in Biofilm Triggers RcsB-Dependent Lipid A Palmitoylation in Escherichia coli
Source: mBio. 2018 Aug 21;9(4):e01415-18. doi: 10.1128/mBio.01415-18 (PMC6106083; doi:10.1128/mBio.01415-18)
Supplement: FIG S7 [file mbo004184028sf7.pdf]

A

pH	24h	48h	72h
Pk	6.5-7	6.5-7	6.5-7
Bf	5.5	6-6.5	6-6.5

B

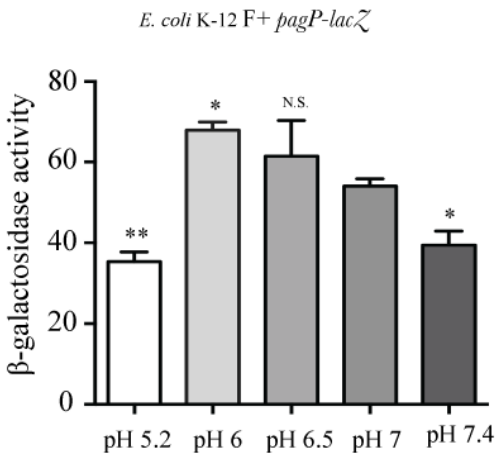

Supplementary Figure S7 Effect of pH variation on *pagP* expression.

(A) *E. coli* K-12 MG1655 F+ was grown under planktonic (Pk) or biofilm (Bf) conditions for 24, 48 and 72 h and pH of the culture medium or biofilm supernanatant was measured using pH indicator strips. **(B)** *E. coli* K-12 MG1655 F+ *pagP-lacZ* was grown overnight in planktonic cultures in M63B1 0.4% glucose adjusted at different pH, and β-galactosidase activity was measured. Statistical significance (compared to the control at pH 7) was assessed using one-way analysis of variance (ANOVA) followed by *Bonferroni's* post-hoc comparison *tests* (* $p < 0.05$; ** $p < 0.01$; NS, not significant).
