# Supplementary material for: Increased Osmolarity in Biofilm Triggers RcsB-Dependent Lipid A Palmitoylation in Escherichia coli
Source: mBio. 2018 Aug 21;9(4):e01415-18. doi: 10.1128/mBio.01415-18 (PMC6106083; doi:10.1128/mBio.01415-18)
Supplement: FIG S8 [file mbo004184028sf8.pdf]

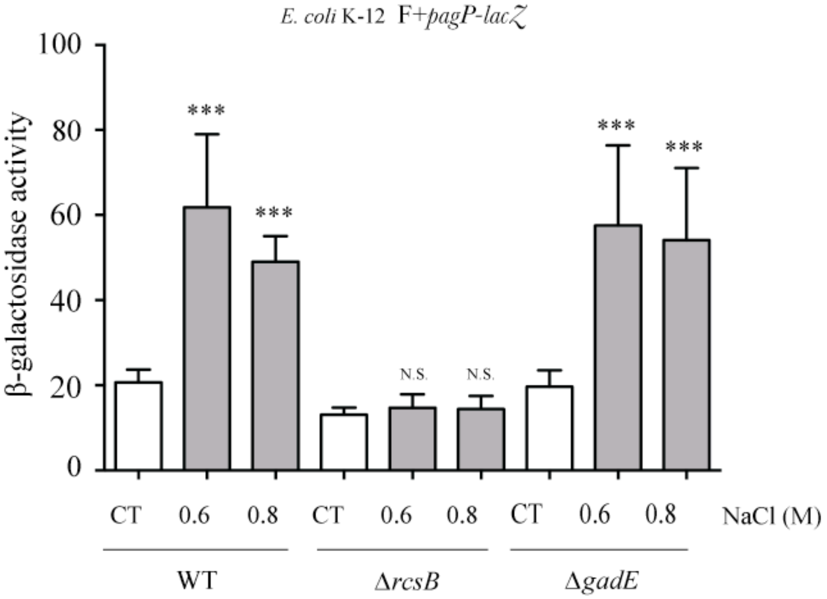

Supplementary Figure S8. NaCl-dependent induction of *pagP* in planktonic cultures does not require GadE.

E. coli K-12 MG1655 F+ *pagP-lacZ*, *E. coli* K-12 MG1655 F+ Δ*rcsB pagP-lacZ, E. coli* K-12 MG1655 F+ Δ*gadE pagP-lacZ* strains were grown in planktonic cultures to OD =0.5 and exposed for 1 h to NaCl (0.6M or 0.8M). β-galactosidase activity was measured and statistical significance was assessed against the corresponding control without NaCl (CT) using one-way analysis of variance (ANOVA) followed by *Bonferroni's* post-hoc comparison *tests* (*** $p < 0.001$; NS, not significant).
