# Supplementary material for: Increased Osmolarity in Biofilm Triggers RcsB-Dependent Lipid A Palmitoylation in Escherichia coli
Source: mBio. 2018 Aug 21;9(4):e01415-18. doi: 10.1128/mBio.01415-18 (PMC6106083; doi:10.1128/mBio.01415-18)
Supplement: FIG S9 [file mbo004184028sf9.pdf]

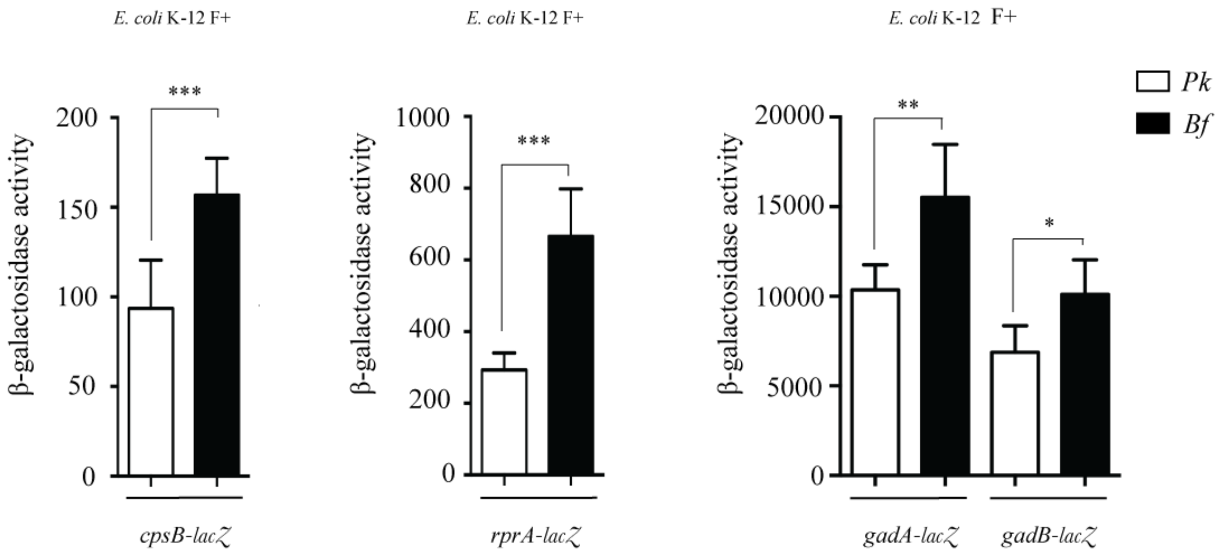

Supplementary Figure S9. *cpsB, rprA* and *gadA/B* are induced in biofilm condition. *E. coli* K-12 MG1655 F+ *cpsB-lacZ*, *E. coli* K-12 MG1655 F+ *rprA-lacZ, E. coli* K-12 MG1655 F+ *gadA-lacZ* and *E. coli* K-12 MG1655 F+ *gadB-lacZ* strains were grown in planktonic (Pk) and biofilm (Bf) for 48 h. β-galactosidase activity was measured. Statistical significance was assessed using one-way analysis of variance (ANOVA) followed by *Bonferroni's* post-hoc comparisons *tests* (* $p < 0.05$; ** $p < 0.01$; *** $p < 0.001$).
