# Supplementary material for: Increased Osmolarity in Biofilm Triggers RcsB-Dependent Lipid A Palmitoylation in Escherichia coli
Source: mBio. 2018 Aug 21;9(4):e01415-18. doi: 10.1128/mBio.01415-18 (PMC6106083; doi:10.1128/mBio.01415-18)
Supplement: TABLE S1 [file mbo004184028st1.pdf]

SUPPLEMENTARY TABLES

Table S1. Strains and plasmids used in this study

Strains and plasmids	Relevant characteristics	References[a]
***Escherichia coli* strains**		
BW25113	*E. coli* K-12 derivative	Keio collection (1)
BW25113 F'*tet*	F' *tet* plasmid in BW25113, biofilm-forming strain; Tet^R	This study
BW25113 *pagP-lacZ*	*lacZzeo* cassette inserted on the chromosome downstream of *pagP* gene; Zeo^R	(2)
BW25113 F'*tet pagP-lacZ*	F' *tet* plasmid in BW25113 *pagP-lacZ;* Zeo^R Tet^R	This study
BW25113 *slyA-lacZ*	*lacZzeo* cassette inserted on the chromosome downstream of *slyA* gene	(2)
BW25113 F'*tet ΔslyA pagP-lacZ*	Δ*slyA::*KmFRT in BW25113 F'*tet pagP-lacZ;* Zeo^R Tet^R Km^R	This study
BW25113 F'*tet ΔrcsB pagP-lacZ*	Δ*rcsB::*KmFRT in BW25113 F'*tet pagP-lacZ;* Zeo^R Tet^R Km^R	This study
BW25113 F'*tet ΔbglJ pagP-lacZ*	Δ*bglJ::*KmFRT in BW25113 F'*tet pagP-lacZ;* Zeo^R Tet^R Km^R	This study
BW25113 F'*tet ΔluxS pagP-lacZ*	Δ*luxS::*KmFRT in BW25113 F'*tet pagP-lacZ;* Zeo^R Tet^R Km^R	This study
BW25113 F'*tet ΔevgA pagP-lacZ*	Δ*evgA::*KmFRT in BW25113 F'*tet pagP-lacZ;* Zeo^R Tet^R Km^R	This study
BW25113 F'*tet ΔrelA pagP-lacZ*	Δ*relA::*KmFRT in BW25113 F'*tet pagP-lacZ;* Zeo^R Tet^R Km^R	This study
BW25113 F'*tet ΔpspF pagP-lacZ*	Δ*psp::*KmFRT in BW25113 F'*tet pagP-lacZ;* Zeo^R Tet^R Km^R	This study
BW25113 F'*tet ΔdegP pagP-lacZ*	Δ*deg::*KmFRT in BW25113 F'*tet pagP-lacZ;* Zeo^R Tet^R Km^R	This study
BW25113 F'*tet ΔbaeR pagP-lacZ*	Δ*baeR::*KmFRT in BW25113 F'*tet pagP-lacZ;* Zeo^R Tet^R Km^R	This study
BW25113 F'*tet ΔphoP pagP-lacZ*	Δ*phoP::*KmFRT in BW25113 F'*tet pagP-lacZ;* Zeo^R Tet^R Km^R	This study
BW25113 F'*tet ΔompR pagP-lacZ*	Δ*ompR::*KmFRT in BW25113 F'*tet pagP-lacZ;* Zeo^R Tet^R Km^R	This study
BW25113 F'*tet ΔrecA pagP-lacZ*	Δ*recA::*KmFRT in BW25113 F'*tet pagP-lacZ;* Zeo^R Tet^R Km^R	This study
BW25113 F'*tet Δfnr pagP-lacZ*	Δ*fnr::*KmFRT in BW25113 F'*tet pagP-lacZ;* Zeo^R Tet^R Km^R	This study
BW25113 F'*tet ΔrpoS pagP-lacZ*	Δ*rpoS::*KmFRT in BW25113 F'*tet pagP-lacZ;* Zeo^R Tet^R Km^R	This study
BW25113 F'*tet ΔcpxR pagP-lacZ*	Δ*cpxR::*KmFRT in BW25113 F'*tet pagP-lacZ;* Zeo^R Tet^R Km^R	This study
BW25113 F'*tet ΔsoxR pagP-lacZ*	Δ*soxR::*KmFRT in BW25113 F'*tet pagP-lacZ;* Zeo^R Tet^R Km^R	This study
MG1655 F' *tet*	*E. coli* K-12 derivative F+, biofilm-forming strain; Δ*lacIZ*; Cm^R Tet^R	(3)
MG1655 F' *tet* Δ*pagP*	Δ*pagP::*KmFRT in MG1655 F'*tet*	This study
MG1655 F' *tet PcL-pagP*	*pagP* with its own RBS sequence placed under the control of the *amp*PcL cassette λpR promoter; *amp*PcL-*pagP* in MG1655 F'*tet* Cm^R Tet^R Amp^R	This study
MG1655 F' *tet PcL-pagP* Δ*rcsB*	Δ*rcsB::*KmFRT in MG1655 F'*tet amp*PcL-*pagP* Cm^R Tet^R Amp^R	This study
MG1655 F' *tet* Δ*rcsB*	Δ*rcsB::*KmFRT in MG1655 F'*tet*	This study
MG1655 F' *tet pagP-lacZ*	*pagP-lacZ* in MG1655 F'*tet; ;* Cm^R Tet^R Zeo^R	This study
MG1655 F'*tet* Δ*rcsB pagP-lacZ*	Δ*rcsB::*KmFRT in MG1655 F'*tet pagP-lacZ;* Cm^R Zeo^R Tet^R Km^R	This study
MG1655 F'*tet* Δ*rcsD pagP-lacZ*	Δ*rcsD543*::Km in MG1655 F'*tet pagP-lacZ;* Cm^R Zeo^R Tet^R Km^R	This study
MG1655 F'*tet* Δ*rcsC pagP-lacZ*	Δ*rcsC::*KmFRT in MG1655 F'*tet pagP-lacZ;* Cm^R Zeo^R Tet^R Km^R	This study
MG1655 F'*tet ΔrcsF pagP-lacZ*	*rcsF::*KmFRT in MG1655 F'*tet pagP-lacZ;* Cm^R Zeo^R Tet^R Km^R	This study
MG1655 F'*tet ΔrcsA pagP-lacZ*	Δ*rcsA::*GB in MG1655 F'*tet pagP-lacZ;* Cm^R Zeo^R Tet^R Km^R	This study
MG1655 F'*tet* Δ*gadE pagP-lacZ*	Δ*gadE::*KmFRT in MG1655 F'*tet pagP-lacZ;* Cm^R Zeo^R Tet^R Km^R	This study
MG1655 F'*tet* Δ*dctR pagP-lacZ*	Δ*dctR::*KmFRT in MG1655 F'*tet pagP-lacZ;* Cm^R Zeo^R Tet^R Km^R	This study
MG1655 F'*tet* Δ*matA pagP-lacZ*	Δ*matA::*KmFRT in MG1655 F'*tet pagP-lacZ;* Cm^R Zeo^R Tet^R Km^R	This study
MG1655 F'*tet* Δ*pta pagP-lacZ*	Δ*pta::*KmFRT in MG1655 F'*tet pagP-lacZ;* Cm^R Zeo^R Tet^R Km^R	This study
MG1655 F'*tet* Δ*ackA pagP-lacZ*	Δ*ackA::*KmFRT in MG1655 F'*tet pagP-lacZ;* Cm^R Zeo^R Tet^R Km^R	This study
MG1655 F'*tet* Δ*pta pagP-lacZ*	Δ*pta::*FRT, flipping cassette from in MG1655 F'*tet pagP-lacZ* Δ*pta::*KmFRT*;* Cm^R Zeo^R Tet^R	This study
MG1655 F'*tet* Δ*pta* /Δ*ackA pagP-lacZ*	Δ*ackA::*KmFRT in MG1655 F'*tet pagP-lacZ* Δ*pta::*FRT*;* Cm^R Zeo^R Tet^R Km^R	This study
MG1655 F'*tet* Δ*pta* /Δ*ackA*/Δ*rcsC pagP-lacZ*	Δ*rcsC::*KmFRT in MG1655 F'*tet pagP-lacZ* Δ*pta::*FRT Δ*ackA::*FRT*;* Cm^R Zeo^R Tet^R Km^R	This study
MG1655 F'*tet* Δ*trkA pagP-lacZ*	Δ*trkA::*KmFRT in MG1655 F'*tet pagP-lacZ;* Cm^R Zeo^R Tet^R Km^R	This study
MG1655 F'*tet* Δ*kdpD pagP-lacZ*	Δ*kdpD::*KmFRT in MG1655 F'*tet pagP-lacZ;* Cm^R Zeo^R Tet^R Km^R	This study
MG1655 F'*tet* Δ*mscL pagP-lacZ*	Δ*mscL::*KmFRT in MG1655 F'*tet pagP-lacZ;* Cm^R Zeo^R Tet^R Km^R	This study
MG1655 F'*tet* Δ*mscS pagP-lacZ*	Δ*mscS::*KmFRT in MG1655 F'*tet pagP-lacZ;* Cm^R Zeo^R Tet^R Km^R	This study
MG1655 *rcsBD56E* Δ*yfaL*	Δ*yfaL::*KmFRT in MG1655 *rcsBD56E (NM 696)* Km^R	N. Majdalani
MG1655 *rcsBD56S* Δ*yfaL*	Δ*yfaL::*KmFRT in MG1655 *rcsBD56S (NM 696)* Km^R	N. Majdalani
MG1655 F'*tet rcsBD56E pagP-lacZ*	Δ*yfaL/ rcsBD56E* in MG1655 F'*tet pagP-lacZ;* Cm^R Zeo^R Tet^R Km^R	This study
MG1655 F'*tet rcsBD56S pagP-lacZ*	Δ*yfaL/ rcsBD56S* in MG1655 F'*tet pagP-lacZ;* Cm^R Zeo^R Tet^R Km^R	This study
MG1655 F'*tet rcsBD56E rprA-lacZ*	Δ*yfaL/ rcsBD56E* in MG1655 F'*tet rprA-lacZ;* Cm^R Tet^R Km^R	This study
MG1655 F'*tet rcsBD56S rprA-lacZ*	Δ*yfaL/ rcsBD56S* in MG1655 F'*tet rprA-lacZ;* Cm^R Tet^R Km^R	This study
MG1655 F' *tet pagP-lacZ Δhns*	Δ*hns*::FRT, flipping cassette from MG1655 F' *tet pagP-lacZ* Δ*hns*::kmFRT Cm^R Zeo^R Tet^R	This study
MG1655 F' *tet pagP-lacZ ΔrcsB/ Δhns*	Δ*rcsB::*KmFRT in MG1655 F'*tet pagP-lacZ* Δ*hns*::FRT; Cm^R Zeo^R Tet^R Km^R	This study
MG1655 F' *tet cpsB-lacZ*	*cpsB::KmGB-lacZ* in MG1655 F'*tet pagP-lacZ;* Cm^R Tet^R Km^R	This study
MG1655 F' *tet gadA-lacZ*	*trpDC::putPA1303-km-gadA::lacZ* in MG1655 F'*tet*	(4)
MG1655 F' *tet gadB-lacZ*	*trpDC::putPA1303-km-gadA::lacZ* in MG1655 F'*tet*	(4)
MG1655 F' *tet rprA-lacZ*	Lambda phage transduction of *rprA-lacZ* in MG1655 F'*tet*	N. Majdalani (4)
DH368 *ΔrcsD*	Δ*rcsD543*::Km; Km^R	N. Majdalani (5)

**Plasmids**		
pCA24N	Cm^R	(6)
pCA24N-*rcsB*	Cm^R , IPTG-inducible	(6)
pCP20	Plasmid bearing the *flp* recombinase gene; 30°C replication; Cm^R Amp^R	(7)

[a] Bacterial strains from our laboratory collection unless otherwise specified.

SUPPLEMENTARY TABLE 1 REFERENCES

1. Baba T, Ara T, Hasegawa M, Takai Y, Okumura Y, Baba M, Datsenko KA, Tomita M, Wanner BL, Mori H. 2006. Construction of Escherichia coli K-12 in-frame, single-gene knockout mutants: the Keio collection. Molecular systems biology 2:2006 0008.10.1038/msb4100050: 10.1038/msb4100050
2. Chalabaev S, Chauhan A, Novikov A, Iyer P, Szczesny M, Beloin C, Caroff M, Ghigo JM. 2014. Biofilms formed by gram-negative bacteria undergo increased lipid a palmitoylation, enhancing in vivo survival. mBio 5.10.1128/mBio.01116-14: 10.1128/mBio.01116-14
3. Ghigo JM. 2001. Natural conjugative plasmids induce bacterial biofilm development. Nature 412:442-445.10.1038/35086581: 10.1038/35086581
4. Castanie-Cornet MP, Treffandier H, Francez-Charlot A, Gutierrez C, Cam K. 2007. The glutamate-dependent acid resistance system in Escherichia coli: essential and dual role of the His-Asp phosphorelay RcsCDB/AF. Microbiology 153:238-246.10.1099/mic.0.29278-0: 10.1099/mic.0.29278-0
5. Majdalani N, Heck M, Stout V, Gottesman S. 2005. Role of RcsF in signaling to the Rcs phosphorelay pathway in Escherichia coli. Journal of bacteriology 187:6770-6778.10.1128/JB.187.19.6770-6778.2005: 10.1128/JB.187.19.6770-6778.2005
6. Kitagawa M, Ara T, Arifuzzaman M, Ioka-Nakamichi T, Inamoto E, Toyonaga H, Mori H. 2005. Complete set of ORF clones of Escherichia coli ASKA library (a complete set of E. coli K-12 ORF archive): unique resources for biological research. DNA research : an international journal for rapid publication of reports on genes and genomes 12:291-299.10.1093/dnares/dsi012: 10.1093/dnares/dsi012
7. Cherepanov PP, Wackernagel W. 1995. Gene disruption in Escherichia coli: TcR and KmR cassettes with the option of Flp-catalyzed excision of the antibiotic-resistance determinant. Gene 158:9-14
